# Identification of Pelvic Congestion Syndrome Using Transvaginal Ultrasonography. A Useful Tool

**DOI:** 10.3390/tomography8010008

**Published:** 2022-01-04

**Authors:** Irene Valero, Rocio Garcia-Jimenez, Pamela Valdevieso, Jose A. Garcia-Mejido, Jose V. Gonzalez-Herráez, Irene Pelayo-Delgado, Ana Fernandez-Palacin, Jose A. Sainz-Bueno

**Affiliations:** 1 Department of Obstetrics and Gynecology, Valme University Hospital, 3441014 Seville, Spain; irevalarr@gmail.com (I.V.); rociogarji@gmail.com (R.G.-J.); Joseantoniosainz@hotmail.es (P.V.); jagmejido@hotmail.com (J.A.G.-M.); 2 Department of Obstetrics and Gynecology, University of Seville, 3441014 Seville, Spain; 3 Department of Angiology and Vascular Surgery, Valme University Hospital, 3441014 Seville, Spain; jvgonzalezherraez@hotmail.es; 4 Department of Obstetrics and Gynecology, Ramón y Cajal University Hospital, 3428042 Madrid, Spain; ipelayod@yahoo.com; 5 Department of Obstetrics and Gynecology, University of Alcalá de Henares, 3428803 Madrid, Spain; 6 Biostatistics Unit, Department of Preventive Medicine and Public Health, University of Seville, 3428803 Seville, Spain

**Keywords:** pelvic congestion syndrome, chronic pelvic pain, transvaginal ultrasound, venography

## Abstract

The gold standard for the diagnosis of pelvic congestion syndrome (PCS) is venography (VG), although transvaginal ultrasound (TVU) might be a noninvasive, nonionizing alternative. Our aim is to determine whether TVU is an accurate and comparable diagnostic tool for PCS. An observational prospective study including 67 patients was carried out. A TVU was performed on patients, measuring pelvic venous vessels parameters. Subsequentially, a VG was performed, and results were compared for the test calibration of TVU. Out of the 67 patients included, only 51 completed the study and were distributed in two groups according to VG results: 39 patients belonging to the PCS group and 12 to the normal group. PCS patients had a larger venous plexus diameter (15.1 mm vs. 12 mm; *p* = 0.009) and higher rates of crossing veins in the myometrium (74.35% vs. 33.3%; *p* = 0.009), reverse or altered flow during Valsalva (58.9% vs. 25%; *p* = 0.04), and largest pelvic vein ≥ 8 mm (92.3% vs. 25%). The sensitivity and specificity of TVU were 92.3% (95% CI: 78.03–97.99%) and 75% (95% CI: 42.84–93.31%), respectively. In conclusion, transvaginal ultrasonography, with the described methodology, appears to be a promising tool for the diagnosis of PCS, with acceptable sensitivity and specificity.

## 1. Introduction

Pelvic congestion syndrome (PCS) is one of the existing causes of chronic pelvic pain (CPP) in women, which consists of the dilation and stasis of the pelvic venous plexus. CPP is defined as the noncyclical pain in the hypogastric, lumbosacral, or perineal area, which lasts 6 months or longer. It is quite an important health problem that causes up to 40% of referrals for gynecology units [1,2].

Currently, the diagnosis of PCS remains a challenge, given that there are not universally accepted criteria for enlarged pelvic venous vessels. In recent years, the Symptoms–Varices–Pathology (SVP) classification has been published for pelvic venous disorders, encompassing three domains: symptoms, varices, and pathophysiology, while also including the anatomy of abdominal and pelvic veins associated with hemodynamic anomalies and etiology. This instrument, once validated, could help to obtain homogeneous study groups with unified diagnostic criteria [3].

In addition, it is estimated that up to 15% of women between the ages of 20 and 50 years have varicose pelvic veins, although not all of them are symptomatic [4,5,6]. Most authors claim that the gold standard for the diagnosis of PCS is venography (VG) [2,7,8,9]. However, in recent years, some authors have proposed the use of computerized axial tomography (CT), magnetic resonance imaging (MRI), or transabdominal and transvaginal ultrasonography as alternatives [7,10,11]. CT and MRI imaging allow for an extensive view of the various causes of pelvic venous dilation, allowing us to use the SVP classification while also being able to detect additional findings such as the nutcracker syndrome or May–Thurner syndrome, which have therapeutic implications [3,12]. A recent systematic review on the noninvasive diagnostic tools for PCS concluded that, although CT is accessible and fast technique, there are no studies available on its diagnostic value for PCS. Moreover, its ionizing properties are a disadvantage in terms of studying a population of mostly young women of fertile age. Regarding the MRI presents a high sensitivity raging between 88 and 100% while also allowing for the differential diagnosis with some other diseases such as endometriosis. However, the lack of standardized criteria regarding PCS, along with the small sample size in noncontrolled studies, and the limited availability in most clinics, makes it difficult for its recommendation [8].

In comparison with other techniques, transvaginal ultrasound (TVU) is an easily accessible, nonionizing, and minimally invasive technique. It allows for the measurement of pelvic veins and blood flow identification in real time [13]. The main disadvantage is that it requires the examiner to possess some experience, alongside the difficulty involved in properly identifying the ovarian veins. Several parameters have been proposed for the ultrasonographic diagnosis of PCS, such as the dilation of ovarian veins, low blood flow velocity in the ovarian vein, or a dilated arcuate vein in the myometrium communicating to pelvic varicosities [14]. Color Doppler imaging is essential to differentiate between cystic images from varicose veins. It also allows for the identification of reverse or abruptly interrupted flow during Valsalva, which is associated with the backflow or stasis of blood, respectively, identified in ovarian venography [4].

However, there still is some controversy regarding the reference values for the diameter of ovarian veins, with the pathological cut-off raging between 5 and 8 mm. In this study, we aim to determine whether transvaginal 2D and Doppler ultrasonography are useful and reliable diagnostic tools for PCS in comparison with venography, as well as to establish ultrasound parameters for said purpose.

## 2. Materials and Methods

An observational and cross-sectional prospective study was carried out in the Gynecological Ultrasound Unit at the Valme University Hospital between November 2018 and July 2020. Patients were consecutively recruited, including all patients referred by the Pelvic Floor, Gynecology and Vascular Surgery Units, that met the inclusion criteria. The study was approved by Andalucia’s board of biomedicine ethics committee (Code 1314/2017). All participants gave informed consent.

### 2.1. Subjects

The patients included in the study were distributed in two groups depending on whether the subsequent venography results confirmed the presence of PCS. The inclusion criteria were women presenting noncyclical pelvic pain for 6 months or longer. The exclusion criteria were the patient’s refusal to undergo a venography.

### 2.2. Data Collection

For the collection of epidemiological data, the patients completed a questionnaire form prior to the diagnostic tests. These parameters were: age; parity; maximum newborn birth weight; menopausal stage; age of the onset of symptoms; worsening of symptoms after pregnancy; presence of vulvar varicosities during pregnancy; prior medical history of endometriosis, adenomyosis, urologic, or gastrointestinal disorders; presence of varicosities in lower extremities; prior pelvic surgery; presence of uterine fibroids; and the presence of varicosities in the vulva, perineum, buttocks, or lower extremities. In addition, patients stated the levels of pain they experienced in the following cases: walking, sitting, in the supine position, dysmenorrhea, dyspareunia, postcoital pain, and lumbar pain. Level of pain was considered clinically significant when the VAS score was 7 or higher.

### 2.3. Ultrasound Assessment

Ultrasonography assessment was performed by an expert examiner with more than 15 years of experience in gynecological ultrasonography, using a Canon Aplio 500 (Toshiba Medical systems Corp., Tokyo, Japan) with a 6.5 MHz probe. Patients were placed in the gynecological position right after urinating in order to perform the assessment with an empty bladder, as is usual procedure for gynecological ultrasonography assessment.

The ultrasound evaluation began with the complete evaluation of the uterus in the longitudinal and cross-sectional planes, followed by the assessment of the adnexa. Uterine and ovarian volumes were calculated using the simplified formula for prolate ellipsoid [15]. Consecutively, we proceeded to identify the largest pelvic vein (after tracking the uterine vein from its origin at the internal cervical os to the internal iliac veins and collateral branches) along with the complete venous plexus, measuring their anteroposterior diameter in a cross-sectional plane. Subsequently, color and spectral Doppler was used to identify the flow direction and whether there was a change in the flow velocity waveform during Valsalva. The same procedure was repeated for the contralateral side. Afterwards, in a cross-sectional plane of the uterus, color Doppler was used to identify the presence of crossing veins in the myometrium, measuring its maximum anteroposterior diameter.

Therefore, the parameters collected in the ultrasound assessment were uterine volume, right and left ovarian volume, presence of polycystic ovaries (PCO), inner diameter of the largest pelvic vein (right and left side), maximum diameter of the largest venous plexus (right and left side), reverse or altered flow during Valsalva, presence of crossing veins in the myometrium, and maximum diameter of crossing veins in the myometrium. The ultrasonography diagnosis of PCS was based on the established cut-off point of 8 mm diameter for the largest pelvic venous vessel.

After the ultrasound assessment, patients underwent a pelvic venography, performed by specialists in angiology and vascular surgery. The diagnosis of PCS was based on the presence of the dilation of ovarian veins ≥ 10 mm, congestion and/or valvular insufficiency [16,17]. If the diagnosis was confirmed, embolization was made during the same procedure in all 39 patients who were diagnosed with PCS.

### 2.4. Statistical Analysis

Statistical analysis was performed with the statistics software IBM SPSS version 22 (IBM, Armonk, NY, USA). Numeric variables were described as mean and standard deviations for normally distributed variables, while median and interquartile range were used for non-normally distributed variables. Percentages were used for qualitative variables. Normality of the data was made with the Shapiro–wilk test. Comparisons of numeric variables was evaluated using Student’s *t*-test in case of normally distributed data, while the Mann–Whitney U-test was used for non-normally distributed data. The Chi-square test was used for comparisons of qualitative variables. Statistical significance was set at *p* < 0.05.

## 3. Results

A total of 67 patients who met the inclusion criteria were invited to participate in the study. Out of the 67 patients included, only 51 completed the study and were distributed in two groups according to the venography results: 39 patients belonging to the PCS group (PCSG) and 12 to the normal group (NG).

There were no statistically significant differences between study groups regarding epidemiological data. The mean age of patients was 44.85 ± 9.92 and 41.5 ± 6.95 years in the PCSG and NG, respectively, with most women being multiparous (74.3% PCSG; 91.6% NG). Only 11.8% of all patients were menopausal, as were 7.69% of the PCS patients. The mean age of the on-set of symptoms were 37 years in the PCSG and 31 years in the NG. Around 60% of PCS patients had vulvar varicosities during pregnancy, with worsening of the symptoms after pregnancy in 70% of them, which differs from the NG, in which only 50% of patients experienced said worsening of symptoms. The rest of the epidemiological variables showed similar results in both groups, with no statistically significant differences, as can be seen in Table 1.

Regarding ultrasound variables, results are shown in Table 2. There were statistically significant differences in the presence of reverse or altered flow during Valsalva (58.9% PCSG vs. 25% NG; *p* = 0.04) and crossing veins in the myometrium (74.35% PCSG vs. 33.3% NG; *p* = 0.009). There were no differences between groups in the diameters of right or left venous plexus, although, when comparing the diameters of the largest venous plexus with both sides combined, we found that PCS patients had a larger venous plexus diameter than normal patients (15.1 mm vs. 12 mm; *p* = 0.009). The presence of any pelvic vein of ≥8 mm was also significatively higher in PCS patients (92.3% vs. 25%; *p* < 0.000). None of the rest of ultrasound variables reached statistical significance.

We also evaluated whether there were discrepancies in parameters based on laterality, given the anatomical differences for venous return. We found that the largest pelvic vein on the left side had a larger diameter than the one on the right side (6 mm vs. 4.5 mm; *p* = 0.011). Ovarian volume and the venous plexus diameter did not reach statistical significance, as can be seen in Table 3.

For the evaluation of the TVU diagnostic accuracy of PCS, in comparison with the diagnostic gold standard pelvic venography (Table 4), we established the TVU diagnostic criteria as the presence of any pelvic vein of 8 mm diameter or larger. In Table 5, we can see that TVU, using this cut-off point, had a sensitivity and specificity of 92.31% and 75%, respectively, with false positive and false negative rates of 7.69% and 25%. Positive and negative predictive values were 92.28% and 75.07%, respectively, for a PCS prevalence of 76.4%.

In addition, a secondary analysis was performed to evaluate whether factors known to increase pelvic veins diameters (parity, premenopause, adenomyosis, fibroids, and PCO) were associated with larger pelvic veins or venous plexus diameters. No statistically significant differences were observed in this regard (Table 6).

## 4. Discussion

It is difficult to know the real incidence of PCS given the lack of definitive diagnostic criteria. It is estimated that the prevalence of PCS, although underdiagnosed, ranges between 16 and 30% of CPP patients in whom other causes have been discarded [18]. In our study, PCS prevalence was 76.4%. This discrepancy might be explained by the fact that our patients were referred after a directed anamnesis and examination by a specialist in angiology and vascular surgery, which might have caused some selection bias.

This disorder is usually diagnosed in multiparous women aged between 30 and 40 years, who suffer a worsening of symptoms after pregnancy, usually with the onset of vulvar varicosities during said period. The explanation might lie in the increase of up to 50% of the pelvic venous vessels’ capacity during pregnancy, which would lead to venous insufficiency and backflow after pregnancy [2,4,18]. In our study, the mean age of PCS patients was 41 years, while the on-set of symptoms was at 31 years. This 10-year delay could be based on the unawareness of this disorder by both patients and medical professionals. Pelvic venous insufficiency is frequent in multiparous women due to major strains on the venous system during pregnancy. Despite this, we did not find an association between PCS and multiparity, varicose veins during pregnancy, or a worsening of symptoms after pregnancy, although the latter was 20% more frequent in the PCS group [19].

Typically, PCS is considered a specific condition of premenopausal women, which is explained by the decrease in estrogen and its vasodilator effect in menopause. This explanation is backed by the improvement patients experience after pharmacological or surgical induction of a hypoestrogenic state [20]. Nonetheless, the etiology and physiology of PCS is multifactorial, thus we did not consider menopause as an exclusion criterion, with 7.69% of PCS patients being in menopausal stage.

The most frequent symptoms of PCS are dysmenorrhea (84%), dyspareunia, and postcoital pain (40.8%), which are usually accompanied by varicosities in the vulva (45.9%) or in the lower extremities (58.7%) [18]. Our results are similar to this data, except our dysmenorrhea rate in PCS patients was somewhat lower (58.9%).

It is also worth mentioning that, in our study, we found that pelvic veins on the left side were significantly larger than on the right side, which is in agreement with the established higher prevalence of PCS on the left side, given the existing anatomical differences between both sides [21].

The main controverse in the diagnosis of PCS is the lack of consensus for what should be considered as enlarged pelvic venous vessels. It is worth mentioning that the existence of anatomical variations in the pelvic venous network may increase the difficulty of its standardization and, moreover, the collapse of the varicose veins due to the filling of the bladder when performing a transabdominal ultrasound assessment may increase difficulty in this regard. The mean ovarian vein diameter is 3.8 mm when valves are competent and 7.5 mm if they are incompetent, therefore the cut-off point for dilated pelvic veins was set at 5 mm. Thus, the dilation is considered mild if the diameter is between 5 and 7.9 mm or severe if it is of 8 mm or larger. However, some authors have only used the cut-off point of 5 mm, and even apply the same criterion for any pelvic vein. Given these discrepancies, a comparison of existing articles remains challenging [20,22]. Even more, whilst ovarian vein imaging is feasibly achievable by venography or tomography, their small size and variable location increase the difficulty of obtaining images by TVU.

Hence, the established criteria in our study for the ultrasonography diagnosis of PCS was the presence of any dilated pelvic vein, with the cut-off point for dilation being 8 mm (severe dilation), with the aim of establishing a reproducible technique.

To date, venography is still considered the *gold standard* for the diagnosis of PCS, despite being an invasive technique that can only be performed in specialized centers, which makes the introduction of new methods, such as ultrasonography, a necessity. A meta-analysis including six studies that compared TVU with venography described sensitivity and specificity rates ranging between 83 and 100% for the identification of pelvic varicocele or ovarian vein ≥ 5 mm. However, it concluded that more studies are needed, given the flawed methodology and heterogeneity of parameters [7,8,10,20,23].

**Figure 1:**
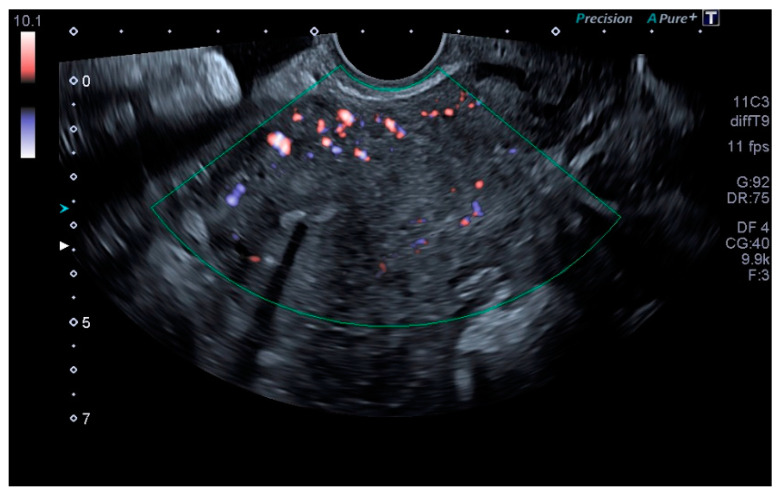
Crossing veins in the myometrium.

**Figure 2:**
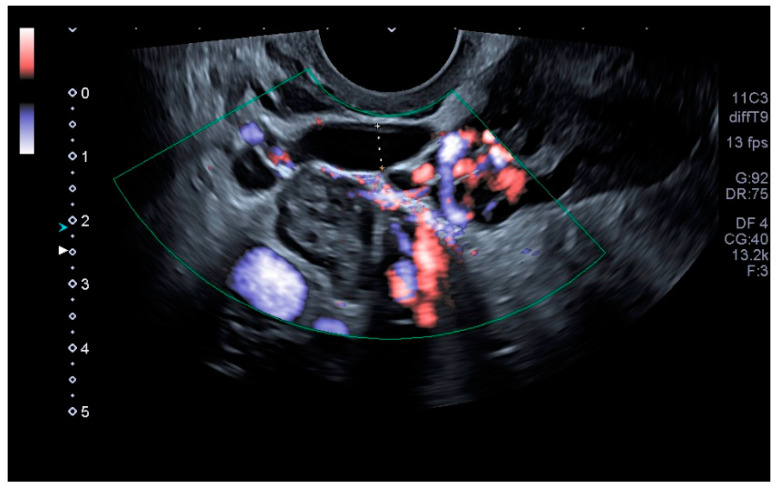
Largest pelvic vein diameter measurement.

**Figure 3:**
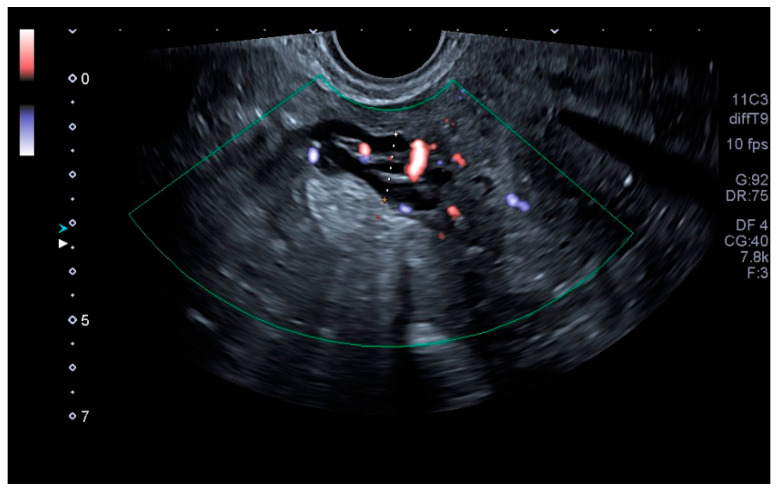
Largest venous plexus diameter measurement.

**Figure 4:**
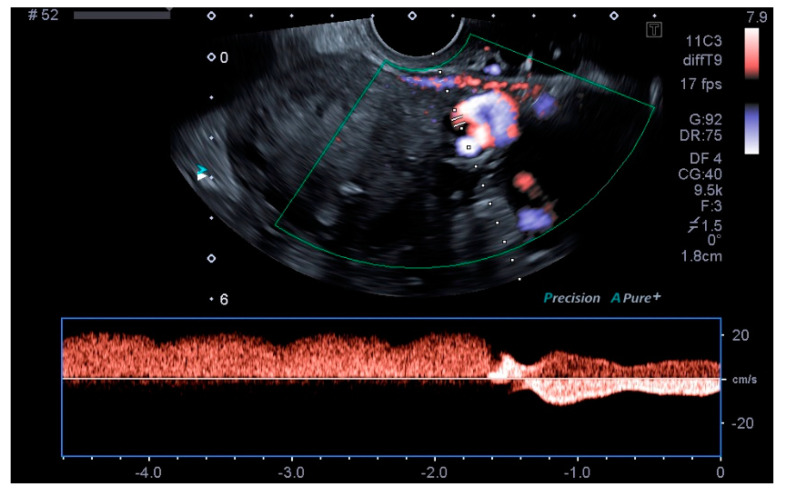
Reverse flow during Valsalva.

**Figure 5:**
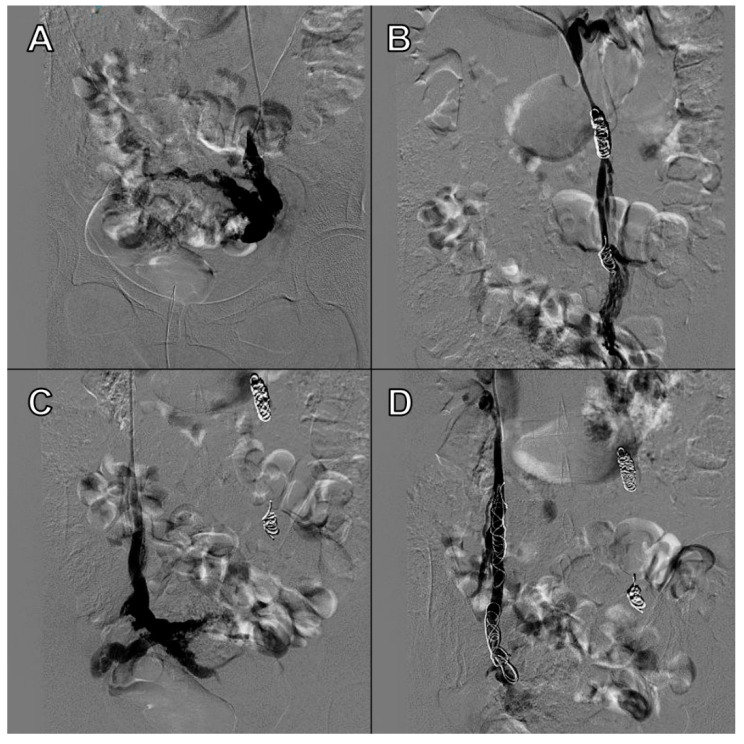
**Venography sequence:** (**A**) Insufficient left ovarian vein with periuterine varices. (**B**) Competent left ovarian vein after embolization. (**C**) Insufficient right ovarian vein with periuterine varices. (**D**) Competent right ovarian vein after embolization.

Our main objective was to determine whether TVU is reliable for the diagnosis of PCS and if it is comparable to venography, as well as to establish ultrasound parameters for said purpose. Our results show that some ultrasound parameters are associated with the PCS, such as a reverse or altered flow during Valsalva, the presence crossing veins in the myometrium, and a larger maximum diameter of pelvic venous plexus. Even more, the ultrasound criteria for the PCS established in this studio (any pelvic vein of ≥8 mm diameter) showed good sensitivity (92.31%) and acceptable specificity (75%), with a low false positive rate (7.69%). Even more, our study showed a high positive predictive value (92.28%) when the PCS prevalence was of 76.4% or, in other words, with clinical suspicion of PCS. Therefore, TVU is a valid alternative for venography that could be used in the first assessment of patients suspected of having PCS. Images of these significant ultrasound variables are displayed in Figure 1, Figure 2, Figure 3 and Figure 4 and Videos S1 and S2. Comparative venography images are shown in Figure 5.

Previous studies have described that the presence of PCO and other factors known to increase uterine volume, such as parity, premenopause, adenomyosis, or fibroids, are associated with larger diameters of pelvic veins. Thus, patients with these characteristics would require different cut-off points [20,22]. In our study, these patients showed no increased venous diameters, hence no different cut-off points were made.

Our study also has its limitations, one of them being the small sample size. It should also be mentioned that the ultrasound evaluation was performed by only one expert examiner in just a single act, and thus we were not able analyzed the intra- and inter-observer reproducibility. We believe that our promising results should be confirmed in future studies, where they would be carried out by nonexpert examiners, and where the inter- and intra-observer reproducibility could be studied. It is also worth mentioning that patients were not required to remain seated or in supine position for a period of time, nor did they fast overnight before the ultrasound assessment, which might have hindered the correct visualization of venous structures due to the interference of intestinal gas. These limitations will be considered in future studies.

## 5. Conclusions

Transvaginal 2D and Doppler ultrasonography, with the described methodology, has acceptable sensitivity and specificity values. Our findings suggest that transvaginal ultrasonography seems to be a promising tool for the diagnosis of pelvic congestion syndrome.

## Figures and Tables

**Table 1:** Epidemiological data.

Variables	PCS Group	Normal Group	All Patients	*p*
Age	41.5 ± 6.95	44.85 ± 9.92	42.2 ± 7.8	0.212
Multiparity	29 (74.3%)	11 (91.6%)	40 (80%)	0.197
Maximum newborn birth weight	3565.56 ± 546.7	3665 ± 442.14	3591.9 ± 518.5	0.476
Menopausal	3 (7.69%)	3 (25%)	6 (11.8%)	0.165
Age of the on-set of symptoms	31.5 ± 8.5	37.25 ± 12.7	32.96 ± 9.9	0.847
Worsening of symptoms during pregnancy	27 (69.2%)	5 (41.6%)	32 (64%)	0.119
Vulvar varicosities during pregnancy	24 (61.5%)	8 (66.6%)	32 (64%)	0.83
Medical history	33 (84.6%)	10 (83.3%)	43 (84.3%)	0.396
Endometriosis	1 (2.5%)	1 (8.3%)	2 (3.9%)	0.449
Adenomyosis	1 (2.5%)	2 (16.6%)	3 (5.9%)	0.156
Urologic disorders	3 (7.69%)	0 (0%)	3 (5.9%)	0.405
Gastrointestinal disorders	0 (0%)	1 (8.3%)	1 (2%)	0.255
Varicosities in lower extremities	27 (69.2%)	6 (50%)	33 (64.7%)	0.105
Prior pelvic surgery	3 (7.9%)	2 (16.6%)	5 (9.8%)	0.378
Fibroids	4 (10.2%)	3 (25%)	7 (13.7%)	0.256
Presence of varicosities (vulva, perineum, buttocks, lower extremities)	29 (74.3%)	7 (58.3%)	36 (70.6%)	0.14
Pain (VAS score ≥ 7)				
Walking	21 (53.8%)	7 (58.3%)	28 (56%)	0.856
Sitting	19 (48.7%)	4 (33.3%)	23 (46%)	0.2
Supine	15 (28.4%)	5 (41.6%)	20 (40%)	0.895
Dysmenorrhea	23 (58.9%)	7 (58.3%)	30 (60%)	0.599
Dyspareunia	16 (41%)	4 (33.3%)	20 (40%)	0.43
Postcoital pain	22 (56.5%)	9 (75%)	31 (62%)	0.532
Lumbar pain	13 (33.3%)	4 (33.3%)	17 (34%)	0.775

PCS: Pelvic congestion syndrome; VAS: Visual Analogue Scale.

**Table 2:** Ultrasound Parameters.

Variables	PCS Group	Normal Group	All Patients	*p*
Uterine volume	80 ± 30.8	73 ± 66.8	79.7 ± 41.4	0.626
Right ovarian volume	9.94 ± 6.2	13.6 ± 13.1	10.65 ± 8	0.514
Left ovarian volume	12.64 ± 8.5	15.2 ± 14.4	13.26 ± 9.9	0.5
PCO	6 (15.8%)	3 (23.1%)	9 (17.6%)	0.552
Largest pelvic vein Ø	6.3 ± 4.5	4.8 ± 1.3	5.9 ± 2.8	0.308
Right side	6.8 ± 12.4	3.9 ± 1.7	5.9 ± 10.5	0.411
Left side	6.1 ± 3.2	4.7 ± 2.1	5.7 ± 2.9	0.187
Largest venous plexus Ø	15.1 ± 6.4	12 ± 5.2	16.9 ± 12.1	0.009
Right side	14.3 ± 7.9	10.5 ± 5.3	13.4 ± 7.4	0.185
Left side	18.4 ± 13.1	9.5 ± 4.3	15.9 ± 12.03	0.155
Reverse of altered flow during Valsalva	23 (58.9%)	3 (25%)	26 (51%)	0.04
Crossing veins in the myometrium	29 (74.35%)	4 (33.3%)	33 (64.7%)	0.009
Crossing veins in the myometrium Ø	3.5 ± 1.99	6 ± 4.2	3.75 ± 2.25	0.141
Pelvic vein Ø ≥ 8 mm	36 (92.3%)	3 (25%)	39 (76.5%)	˂0.000

PCS: Pelvic congestion syndrome; PCO: Polycystic ovaries; Ø: Diameter.

**Table 3:** Evaluation of differences based on laterality.

Variables	Left Side	Right Side	*p*
Ovarian volume	8 ± 15.4	7.4 ± 7.99	0.646
Largest pelvic vein Ø	6 ± 3.6	4.5 ± 1.88	0.011
Largest venous plexus Ø	15.2 ± 8.75	15.5 ± 9.75	0.359

Ø: Diameter.

**Table 4:** Evaluation of the transvaginal ultrasound diagnostic accuracy of pelvic congestion syndrome using the criteria of any pelvic vein diameter of 8 mm or larger.

		Venography (Gold Standard)			
		Normal	PCS	Total	*p*
Transvaginal Ultrasound	Normal	9	3	12	˂0.005
	PCS	3	36	39	
	Total	12	39	51	

PCS: Pelvic congestion syndrome.

**Table 5:** Diagnostic values of transvaginal ultrasound for pelvic congestion syndrome using the criteria of any pelvic vein diameter of 8 mm or larger.

Transvaginal Ultrasound	Value	CI 95%
Sensitivity	92.31%	78.03–97.99%
Specificity	75%	42.84–93.31%
Positive predictive value	92.28%	81.71–96.97%
Negative predictive value	75.07%	49.18–90.36%
False positive rate	7.69%	2.01–21.97%
False negative rate	25%	6.7–57.16%

**Table 6:** Assessment of factors associated with larger diameters of pelvic veins.

Transvaginal Ultrasound	Nulliparous	Multiparous	*p*
Largest pelvic vein Ø	5.6 ± 2.3	6.2 ± 2.9	0.25
Largest venous plexus Ø	16 ± 4.55	17.2 ± 12.6	0.75
	Premenopause	Menopause	*p*
Largest pelvic vein Ø	6.2 ± 2.8	4.9 ± 2.1	0.43
Largest venous plexus Ø	16 ± 7.3	27.5 ± 35.2	0.33
	No adenomyosis	Adenomyosis	*p*
Largest pelvic vein Ø	5.8 ± 2.8	6.9 ± 0.21	0.33
Largest venous plexus Ø	15.9 ± 11.6	-	-
	No fibroids	Fibroids	*p*
Largest pelvic vein Ø	6.1 ± 2.9	6.2 ± 0.61	0.7
Largest venous plexus Ø	17.1 ± 12.4	16.5 ± 5.7	0.17
	Normal ovaries	PCO	*p*
Largest pelvic vein Ø	6.11 ± 2.9	6.2 ± 1.8	0.44
Largest venous plexus Ø	17.7 ± 12.8	14 ± 4.8	0.98

Ø: Diameter.

## Supplementary Materials

The following are available online at https://www.mdpi.com/article/10.3390/tomography8010008/s1, Video S1: Crossing veins in the myometrium, Video S2: Reverse flow during Valsalva.
